# Supplementary material for: Immune and pathophysiologic profiling of antenatal coronavirus disease 2019 in the GIFT cohort: A Singaporean case-control study
Source: Front Pediatr. 2022 Sep 15;10:949756. doi: 10.3389/fped.2022.949756 (PMC9521552; doi:10.3389/fped.2022.949756)
Supplement: Supplementary file 1 [file Data_Sheet_1.pdf]

Supplementary Material

1 Supplementary Data

Supplementary Data 1. Protein BLAST result between S21P2 peptide and HCV proteins.

2 Supplementary Figures and Tables

2.1 Supplementary Figures

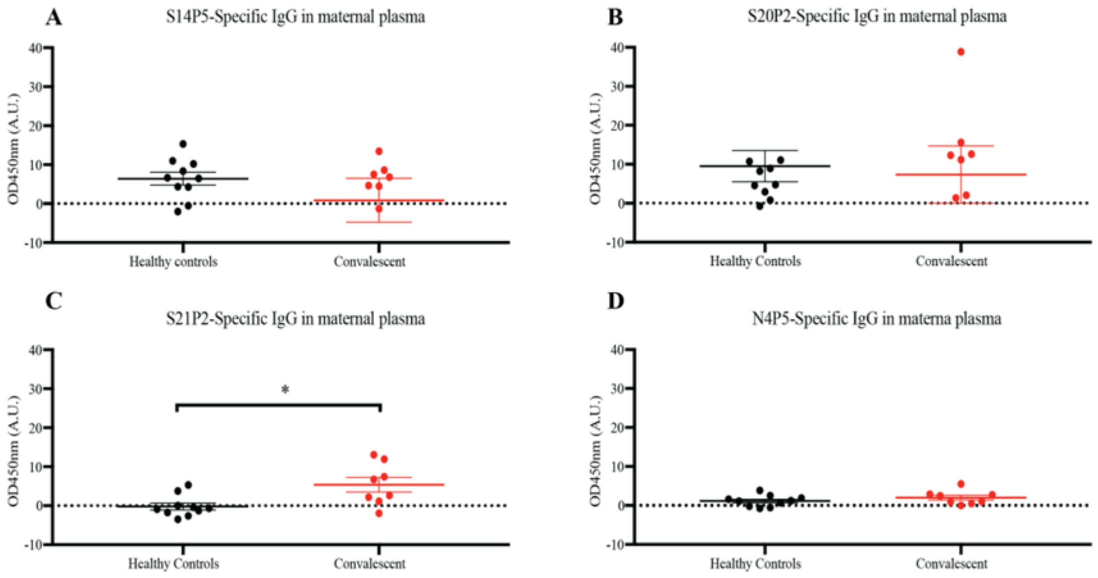

Supplementary Figure 1. Screening IgG antibodies against four immunodominant epitopes in maternal plasma. Using 1-month post-partum maternal plasma from control (CT01-CT10) and convalescent (CS01-CS08) mothers. An evaluation of IgG levels against (A) S14P5, (B) S20P2, (C)S21P2, and (D) N4P5 peptides. Bar graphs represent the normalized average signals, and the dotted line is the averaged signals from the controls.

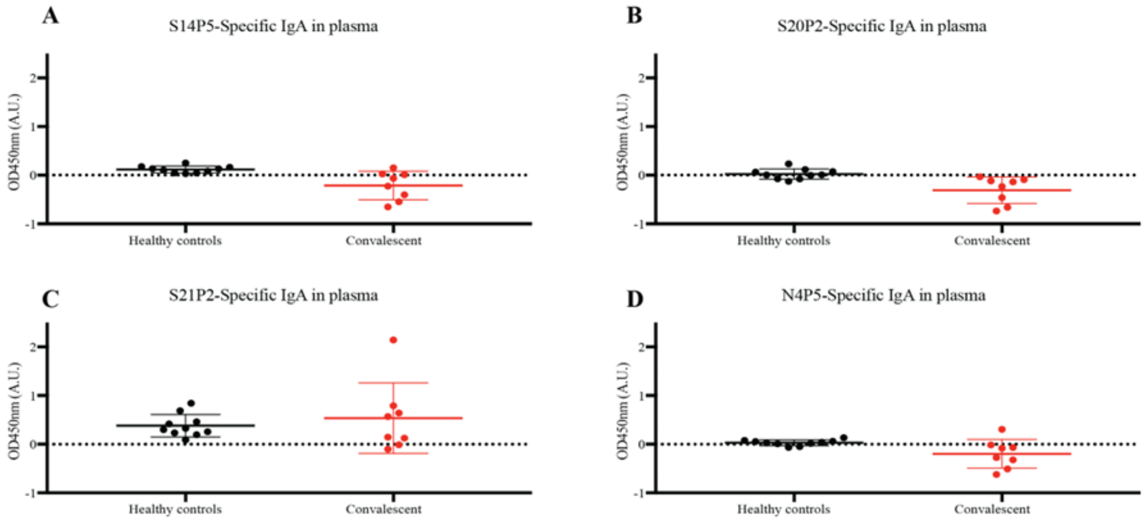

Supplementary Figure 2. Screening IgA antibodies against four immunodominant epitopes in maternal plasma. Using 1-month post-partum maternal plasma from control (CT01-CT10) and convalescent (CS01-CS08) mothers. An evaluation of IgA levels against (A) S14P5, (B) S20P2, (C)S21P2, and (D) N4P5 peptides. Bar graphs represent the averaged normalized signals, and the dotted line is the averaged signals from the controls. The sample with the highest signal in (C) under convalescent mothers represents CS04.

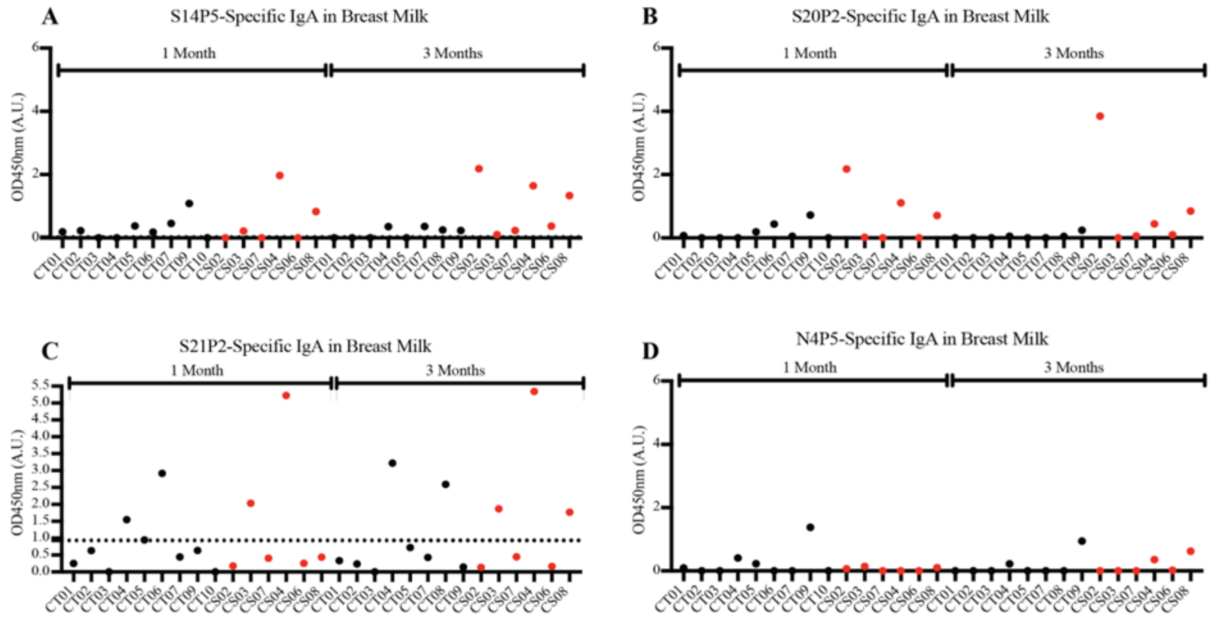

Supplementary Figure 3. Screening IgA antibodies against four immunodominant epitopes in maternal breast milk at 1 and 3 months post-partum. Using 1 and 3-month post-partum maternal breast milk from control (CT01-CT10) and convalescent (CS01-CS08) mothers. An evaluation of IgA levels against (A) S14P5, (B) S20P2, (C) S21P2, and (D) N4P5 peptides. Bar graphs represent the averaged normalized signals, and the dotted line is the averaged signals from the controls. Convalescent mothers (CS samples) are arranged in ascending order according to their time from COVID diagnosis to delivery.

Supplementary Table 1. Information on the time from diagnosis to delivery for the convalescent participants.

Sample	Interval from COVID diagnosis to delivery (Days)
CS01	37
CS02	69
CS03	76
CS04	105
CS05	111
CS06	226
CS07	92
CS08	250

Supplementary Table 2. **Individual characteristics of convalescent women.**

	CS01	CS02	CS03	CS04	CS05	CS06	CS07	CS08
Maternal Age (Years)	30	26	36	35	27	31	27	30
Maternal Ethnicity	Indian	Caucasian	Caucasian	Malay	Malay	Chinese	Indian	Malay
Diagnosis of COVID-19 (Gestation, weeks)	36	32	29	24	22	10	28	4
COVID severity (WHO classification)	Mild	Mild	Mild	Mild	Mild	Moderate	Asympt omatic	Mild
Mode of delivery	Vaginal	Vaginal	Vaginal	Vaginal	Vaginal	Vaginal	Vaginal	Vaginal

Supplementary Table 3. Individual laboratory results of convalescent women peri-delivery.

	CS01	CS02	CS03	CS04	CS05	CS06	CS07	CS08
COVID-19 nasopharyngeal swab (closest swab done prior to delivery)	Negative	Negative	Negative	Negative	Negative	Not done	Not done	Not done
COVID-19 in serum	Negative	Negative	Negative	Negative	Negative	Negative	Negative	Negative
COVID-19 in vaginal swab	Negative	Negative	Not done	Negative	Not done	Negative	Negative	Negative
COVID-19 swab of umbilical cord	Negative	Negative	Negative	Negative	Negative	Negative	Negative	Negative
COVID-19 swab of placenta	Negative	Negative	Negative	Negative	Negative	Negative	Negative	Negative
COVID-19 swab of amniotic fluid	Not done	Negative	Not done	Negative	Negative	Negative	Negative	Not done
COVID-19 swab of breast milk	Not done	Negative	Negative	Negative	Not done	Negative	Inconclusive	Not done

Supplementary Table 4. Individual characteristics of neonates born to convalescent women.

	CS01	CS02	CS03	CS04	CS05	CS06	CS07	CS08
Mode of delivery	Vaginal	Vaginal	Vaginal	Vaginal	Vaginal	Vaginal	Vaginal	Vaginal
Gestational Age (weeks + days)	41+2	39+6	39+4	39+5	38+0	41+3	39+0	40+1
Gender	Male	Female	Female	Female	Female	Male	Female	Female
Birth weight (g)	3915	3210	3895	3505	2695	3525	3645	3750
OFC (cm)	35.5	34	36	33.5	33	35	34	35
Length (cm)	52	48	52	52	47	52	50	51
APGAR score at 1, 5 minutes of life respectively	9,9	9,9	9,9	9,9	9,9	9,9	9,9	9,9
